# Editorial: Advancing gene and cell therapy using human mesenchymal stem cells

**DOI:** 10.3389/fcell.2023.1294460

**Published:** 2023-10-13

**Authors:** Tong Ming Liu, Yingnan Wu

**Affiliations:** ^1^ Institute of Molecular and Cell Biology (IMCB), Agency for Science, Technology and Research (A*STAR), Singapore, Singapore; ^2^ Department of Orthopaedic Surgery, Yong Loo Lin School of Medicine, National University of Singapore, Singapore, Singapore; ^3^ NUS Tissue Engineering Program, Life Sciences Institute, National University of Singapore, Singapore, Singapore

**Keywords:** mesenchymal stem cells, senescence, cell therapy, gene therapy, MSC-EVs

Human mesenchymal stem cells (hMSCs) have emerged as an attractive cell source for regenerative medicine. MSCs are adult stem cells, which reside in almost all adult tissues and birth-associated tissues. As one of the most clinically used stem cells, MSCs have been used in over 1,500 clinical trials to treat over 30 diseases. However, primary MSCs have limited life span, resulting in limited cells (Liu et al., 2013; Liu et al., 2020; Liu, 2021). During *in vitro* expansion, MSCs gradually lose differentiation potential and become senescent, which greatly compromises their therapeutic applications. Gene and cell therapy of MSCs offers a promising treatment option for regenerative medicine. This Research Topic contains a collection of articles exploring MSC senescence, iMSCs from iPSCs, chondrogenesis of iMSCs, and the application of MSCs or MSC-Exos for repairing heart injuries and treating stress urinary incontinence.

MSCs become senescent during *in vitro* expansion. To understand the molecular basis of MSC senescence and identify biomarkers used to evaluate MSC senescence, Yang et al. re-analyzed sequencing data in the public domain and established a regulatory network of “transcription factor (TF)-miRNA-Target” associated with the senescence and quality status of MSCs during passage. This study provides insights into key molecules for evaluating the passage-dependent replicative senescence of MSCs and deciphers the mechanism of miRNAs regulating the senescence of MSCs during *in vitro* expansion. This study provides passage-dependent indicators for monitoring the quality of MSCs during the clinical application.

Induced pluripotent stem cells (iPSCs) can self-renew unlimitedly and differentiated into any type of cells, including MSCs. Therefore iPSC-MSCs (iMSCs) can provide unlimited iPSC-MSCs to overcome limited MSCs with primary MSCs and represent an alternative source to MSCs (Liu et al., 2020; Liu, 2021). However, the use of iPSCs for regenerative medicine has concern over safety due to possible iPSCs contamination in iMSCs causing tumors. In this Research Topic, Al-Akashi et al. used the Brequinar (BRQ) to eradicate the leftover iPSCs and purify iPSC-MSCs. BRQ is a key enzyme in *de novo* pyrimidine biosynthesis, which inhibits dihydroorotate dehydrogenase (DHODH) to prevent the growth of 3D iPSC aggregates. The authors found that BRQ can be used to eradicate the remaining undifferentiated hiPSCs, there were no changes in survival, differentiation potential and gene expression between BRQ-treated iMSCs and non-treated iMSCs, suggesting that BRQ can effectively purify iMSCs from hiPSCs, this will reduce concern over safety of iMSC based therapy. In addition, iMSC quality is critical to clinical application. Using a stepwise protocol to differentiate iPSCs toward MSCs (iMSCs) via the induction of neural crest cells, Zujur et al. compared two types of xeno-free culture media, XSF and T1 media to generate iMSCs from iPSCs. They also reported that TD-198946 increased the size of chondrogenic spheroids and improved chondrogenesis of iMSCs, shown by enhanced chondrogenic genes and stains. TD-198946 is a thienoindazole derivative and has been shown to be a potent chondrogenic factor. Subcutaneous transplantation of chondrogenic spheroids from iMSCs showed that cartilage extracellular matrix increased without a noticeable effect on hypertrophy. This study provides insights into generating good quality of iMSCs for cartilage tissue engineering.

Extracellular vesicles secreted by MSCs (MSC-EVs) have recently been shown as a promising alternative to MSCs for regenerative medicine (Yang et al., 2023). MSC-EVs are extracellular nanovesicles containing bioactive proteins, nucleic acids, lipids, enzymes and metabolites. Compared with the direct use of MSCs, MSC-EVs have advantages in higher safety, lower immunogenicity, lower immune rejection, effortless storage and the ability to cross biological barriers and avoid complications. Zhu et al. reviewed the applications of exosomes derived from MSCs for the repair of heart injuries. MSC exosomes (MSC-Exos) decrease the apoptosis of myocardial cells and fibrosis, prevent the infiltration of inflammatory factors and increase the formation of blood vessels. The authors discussed MSCs exosomes with a specific focus on the role of MSC-Exos miRNA in myocardial injury repair. Non-coding RNAs (NC-RNAs) in MSC-Exos are affected by various pretreatments, such as drugs, chemicals and hypoxia. The authors further discussed the cardiac protective effects of long non-coding RNAs (lncRNAs) in MSC-Exos and underlying molecular mechanisms of MSC-Exos. This review provides valuable insights for advancements in heart tissue engineering research using MSC-EVs.

Stress urinary incontinence (SUI) is the unintentional loss of urine caused by increased abdominal pressure during physical activity, which adversely affects the quality of life of patients. Current available therapies are not effective in all patients, MSCs represent a promising therapy for treating SUI. Liu et al. reviewed MSC-based treatments of SUI and summarized four treatment strategies, including single MSC type therapy, MSC-based combination therapy, MSC secretome treatment, and other factors affecting MSC therapy. This review discussed the molecular mechanisms underlying MSC mediated SUI treatment. Although evidence supports the safety and effectiveness of MSCs or MSC-EVs for treating SUI, there are limitations with current studies, including small sample sizes, most data from animal studies and few from clinical trials, short-term results and unclear mechanism of action, etc. Therefore, more research and clinical trials are needed for a better understanding and optimization of these treatments.
